# Expression of MMP-9 and VEGF in Meningiomas and Their Correlation with Peritumoral Brain Edema

**DOI:** 10.1155/2015/646853

**Published:** 2015-03-03

**Authors:** Joanna Reszec, Adam Hermanowicz, Robert Rutkowski, Grzegorz Turek, Zenon Mariak, Lech Chyczewski

**Affiliations:** ^1^Department of Medical Pathomorphology, Medical University of Bialystok, Ulica Waszyngtona 13, 15-269 Bialystok, Poland; ^2^Department of Neurosurgery, Medical University of Bialystok, Ulica Marii Skłodowskiej-Curie 24A, 15-276 Bialystok, Poland

## Abstract

Meningiomas constitute up to 13% of all intracranial tumors. The predictive factors for meningioma have not been unambiguously defined; however some limited data suggest that the expression of matrix metalloproteinases (MMPs) and vascular endothelial growth factor (VEGF) may be associated with the presence of peritumoral brain edema (PTBE) and worse clinical outcome. The aim of this study was to analyze the expressions of MMP-9 and VEGF in a group of meningiomas of various grades and to study associations between these two markers and PTBE. The study included patients with supratentorial meningiomas. The patients were divided into low- (G1) and high-grade meningiomas (G2 and G3). PTBE was assessed on MRI. The expressions of VEGF and MMP-9 were determined immunohistochemically. The expression of MMP-9 was observed significantly more often in G3 meningiomas than in lower grade tumors. The presence of stage II or III PTBE was associated with a significant increase in MMP-9 expression. The expression of VEGF did not differ across the PTBE stages. Our findings point to a significant role of MMP-9 and VEGF in the pathogenesis of peritumoral brain edema in low- and high-grade meningiomas.

## 1. Introduction

Meningiomas are common primary brain tumors that constitute about 13% of all intracranial tumors. Most meningiomas have a benign biology and are evaluated as G1; however, some have a tendency to recur or even transform into more malignant forms. There is a lack of predictive factors for meningioma; however, the most important are the number of mitotic figures or the presence of necrosis.

Meningiomas can also differ in their clinical presentation, and even histologically benign meningiomas can exhibit peritumoral brain edema (PTBE) [1]. Brain edema, as a major factor that governs clinical management, is responsible for clinical symptoms and may be associated with surgical and postsurgical complications. Various possible causes of edema associated with meningiomas have been reported but most of them do not sufficiently explain its formation [2–4].

In recent years, many studies have focused on biological mechanisms that may explain different tumor behavior and also serve as prognostic biomarkers or even could be a target in therapeutic strategy. Approximately 60% of meningiomas exhibit peritumoral brain edema, which is associated with a worse postsurgical clinical course. Various possible courses of PTBE have been reported which include tumor size, location, histological differentiation, vascular density, pial blood supply, tumor-related blood obstruction, sex hormone receptors, or others [5–8].

Matrix metalloproteinase (MMP) expression in meningiomas has been studied in relation to tumor invasiveness, PTBE, malignancy, or recurrence [2–4]. MMPs are proteolytic enzymes that degrade extracellular matrix components and take part in normal physiological processes of tissue remodeling. One type, MMP-9, has been assigned an important role during angiogenesis and tumor invasion [9]. Matrix metalloproteinase 9 (MMP-9) is able to degrade the extracellular matrix and basement membrane leading to cancer cell invasion and metastasis. Some have associated MMP-9 with the invasion and metastasis of many types of cancers like lung, breast, or colorectal cancer. In meningiomas, MMP-9 expression in tumors has been correlated with peritumoral brain edema and cell proliferation. Also it has been reported that MMP-9 can affect the MAP kinase pathway leading to the activation of cell proliferation. In brain tumors, mainly in gliomas, MMPs have been well studied according to brain glioma invasion. Recently, limited data suggest that the expression of matrix metalloproteinases (MMPs) may be related with the presence of PTBE in meningiomas [10–12].

Angiogenesis is dependent upon the balance between angiogenic and antiangiogenic regulators. Vascular endothelial growth factor (VEGF) has been demonstrated to play a significant role in the stimulation of angiogenesis in many types of cancers, mainly in non-small cell lung cancer or colorectal cancer. However in spite of higher vascularity encountered in high-grade tumors, the correlation between histological grade and VEGF expression in meningiomas is not so clear. Also, contradictory data on the prognosis of the value of VEGF expression on the recurrence risk of these tumors have been reported [9–11, 13].

PTBE formation is widely accepted to be vasogenic. In meningiomas, vascular endothelial growth factor (VEGF) was found to play an important role as both an angiogenic and a vascular permeability-increasing factor that indirectly promote the development of edema. VEGF is a highly specific mitogen for vascular endothelial cells.

The expression of VEGF is potentiated in response to hypoxia, by activated oncogenes and by a variety of cytokines. VEGF induces endothelial cell proliferation, promotes cell migration, and inhibits apoptosis. In vivo, VEGF induces angiogenesis as well as permeabilization of blood vessels and plays a central role in the regulation of vasculogenesis. Deregulated VEGF expression contributes to the development of solid tumors by promoting tumor angiogenesis and to the etiology of several additional diseases that are characterized by abnormal angiogenesis [14, 15].

The aim of our study was to evaluate the expression of MMP-9 and VEGF in a large group of meningiomas of various grades in correlation with the extent of PTBE.

## 2. Materials

We identified 136 patients with histopathological diagnosis of meningiomas obtained in the Department of Neurosurgery of the Medical University of Bialystok in 1995–2013. We retrospectively waived the 10% buffered, formalin fixed, paraffin embedded sections. The patients were divided into two groups followed by histopathological diagnosis of their tumors: low-grade meningiomas (G1) and high-grade meningiomas (G2 and G3) based on the criteria accepted by WHO (based on WHO classification of tumors of the central nervous system (Lyon 2007)).

For each case, PTBE was evaluated in MRI by an experienced radiologist and graded by Steinhoff classification as follows: 0, no edema; I, peritumoral brain edema limited to 2 cm; II, peritumoral brain edema limited to half of the hemisphere; III, more than half of the hemisphere [16].

### 2.1. Immunohistochemical Analysis

Evaluation of vascular endothelial growth factor (VEGF) and metalloproteinase-9 (MMP-9) proteins expression was done using immunohistochemical methods. Following the deparaffinisation and rehydration, epitope retrieval was carried out in the EnVision Flex Target Retrieval Solution (DAKO) in high pH. Endogenous peroxidases were blocked by incubating the sections in methanol and 3% hydrogen peroxidase for 20 minutes. Next, slides were incubated with polyclonal antibody against VEGF protein (DAKO; the antibody labels the VEGF-121, VEGF-165, and VEGF-189 isoforms of vascular endothelial growth factor) in 1 : 100 dilution for 30 minutes at room temperature and against MMP-9 protein (MMP-9 Antibody (C-20): sc-6840 Santa Cruz Biotechnology) in 1 : 100 dilution for 30 minutes at room temperature. Visualization Reagent EnVision Flex (DAKO) was applied for 30 minutes followed by DAB solution for 10 minutes.

The slides were then counterstained with hematoxylin and examined under a microscope. Immunohistochemical evaluation of each protein expression was performed by two independent pathologists. The intensity of immunostaining was evaluated in 10 random fields under 20x magnification. VEGF and MMP-9 expression was qualitatively scored as follows: 0 for no staining of labeled cells or for a trace of positive cells (less than 10% of cells were labeled), 1+ for moderately diffuse staining or sparsely intensive staining (10%–50% of cells were labeled), and 2+ for strongly diffuse staining (more than 50% of cells were labeled).

Appropriate positive and negative controls were performed. Negative controls were done using a nonimmunized IgG replacing the primary antibody. Known MMP-9 proteins expressed in rheumatoid arthritis, the inflamed synovial specimens, were used as positive controls. Clear cell cancer of the kidney was used as a positive control for VEGF expression. The results were evaluated and compared between all of the groups.

### 2.2. Statistical Analysis

Database management and analysis were performed using statistical software (SYSTAT 12 for Windows). Relationships between the groups were evaluated by the Kruskal-Wallis analysis of variance on ranks. Values of *P* < 0.05 were considered statistically significant.

## 3. Results

In the examined group, we had 136 patients (56 male and 80 female) with meningiomas of various grades: 93 with low grade (G1) and 43 with high grade (G2 and G3). In the group of low-grade meningiomas, 10 were meningiomastous, 10 fibrous, 8 psammomatous, 2 angiomatous, 1 secretory, and 62 mixed type (transitional, Figures 3(a)–3(d)). Patients' age ranged from 28 to 84 years. All of the meningiomas were intracranial, supratentorial tumors. In the group of low-grade meningiomas, all were resected totally; however, in 15 cases we observed features of invasion of the meninges (in 3 of the fibrous and 12 of mixed type).

17 cases recurred and 13 transformed into more malignant tumors within the period of 2000–2013.

### 3.1. MMP-9 and VEGF Expression in Correlation with Meningioma Grade

**Figure 1:**
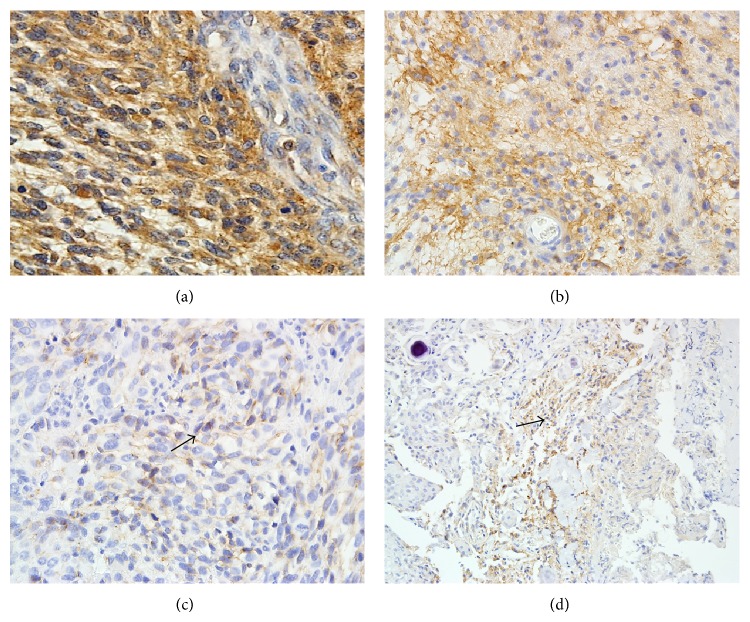
(a) A significant MMP-9 expression in anaplastic meningioma with PTBE III°. Magnification 400x. ((b), (c)) Strong (b) and weak (c) MMP-9 expression in atypical meningioma. Magnifications 200x and 400x. (d) MMP-9 expression in mixed type of low-grade meningioma. Magnification 200x.

In the low grade meningioma group, MMP-9 expression was observed in 65 out of 93 cases (69.9%), mostly as weak expression estimated as (+). The most intense and diffuse MMP-9 expression was observed in tumors with meninges invasion or tumors which recurred (Figure 1(d)).

In the high-grade meningioma group, 40 out of 43 cases (93.1%) presented MMP-9 expression. In atypical meningiomas, the MMP-9 expression was strong (Figure 1(b)) and some showed weak MMP-9 expression (Figure 1(c)). The most significant expression was observed in G3 meningiomas (Figure 1(a)).

**Figure 2:**
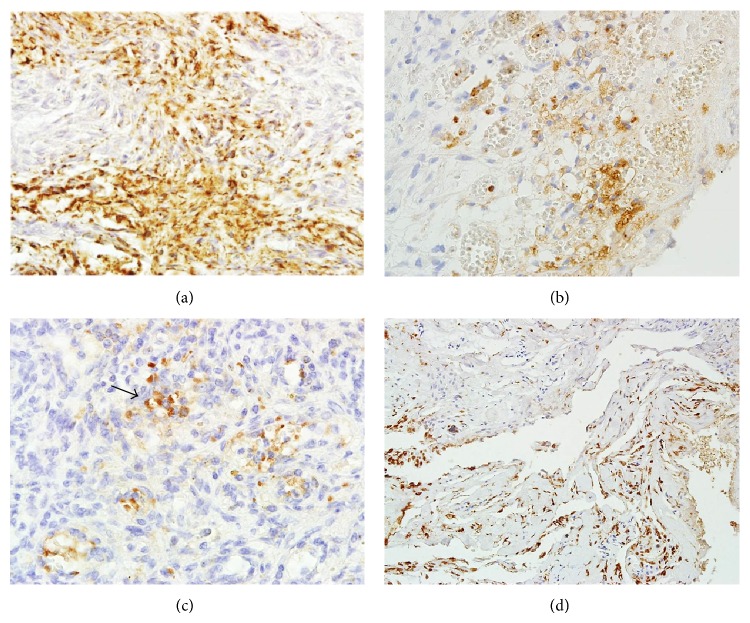
(a) VEGF expression in high-grade meningioma. Magnification 200x. (b) Strong VEGF expression in angiomatous low-grade meningioma. Magnification 400x. (c) VEGF expression in atypical meningioma with peritumoral brain edema II°. Magnification 200x. (d) Mixed type of meningioma with significant VEGF expression and presence of PTBE. Magnification 100x.

**Figure 3:**
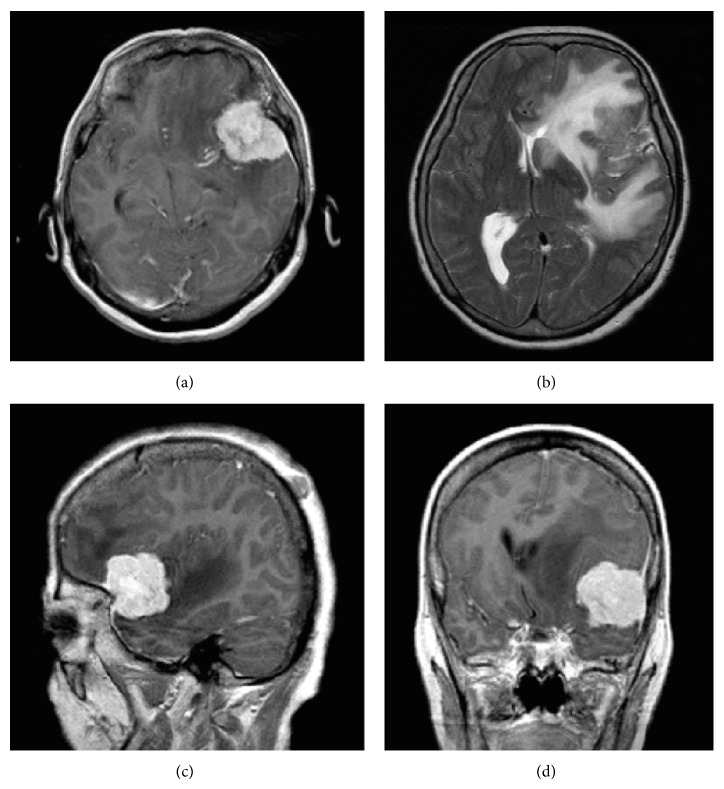
(a)–(d) MRI scans show transitional meningioma (G1) with peritumoral brain edema III°.

VEGF expression was observed in 28 out of 93 low-grade meningiomas (30.1%), mainly in angiomatous (Figure 2(b)), secretory, and mixed types. VEGF expression was positive in 29 out of 43 high-grade meningiomas (67.4%). Also, the most intense VEGF expression was observed in meningiomas which recurred and in anaplastic meningiomas (G3) (Figure 2(a)).

We observed a statistically significant correlation between MMP-9, VEGF expression, and tumor grading (*P* = 0.003 and *P* = 0.001) (Table 2).

### 3.2. MMP-9 Expression in Correlation with Peritumoral Brain Edema (PTBE)

Peritumoral brain edema was observed in 92 out of 136 meningiomas: 49 cases in I stage, 41 in II stage, and 2 in III stage.

Peritumoral brain edema was present in 56 out of 93 low-grade meningiomas, 23 tumors being evaluated as significant (II and III grades in Steinhoff classification). In high-grade meningiomas, peritumoral brain edema was present in 36 out of 43 tumors, 20 being significant (II and III grades in Steinhoff classification). The results are shown in Table 1.

MMP-9 expression was significant mainly in meningiomas with PTBE evaluated as II or III stage in Steinhoff classification (38 and 2 cases, resp.). The result was statistically significant (*P* = 0.028) (Table 3).

VEGF expression was observed in 27 meningiomas with I stage of PTBE (Figure 2(d)), in 17 with II stage, and in 2 with III stage (Figure 2(c)). The result was also statistically significant (Table 4).

## 4. Discussion

Meningiomas are mostly benign brain tumors; however, they have a tendency to recur or even sometimes transform into more malignant tumors—atypical or anaplastic with brain invasion. The clinical course of meningiomas is also associated with the presence of peritumoral brain edema (PTBE), which often prolongs the postsurgical outcome. The pathogenesis of PTBE is still unknown; however, we know that this is a vasogenic type of edema. Our previous study showed a significant correlation between HIF-1*α* and mast cell tryptase expression and PTBE.

There are only a few predictive factors for meningiomas; the most important are mitotic activity, presence of necrosis, and cytologic atypia. In our present study, we tried to determine whether MMP-9 and VEGF may have an influence on peritumoral brain edema formation. In the study, we showed significant MMP-9 expression in both low- and high-grade meningiomas; however, the most significant MMP-9 expression was observed in atypical and anaplastic meningiomas. Furthermore, meningiomas which recurred or transformed into more malignant forms presented high MMP-9 expression.

Some studies confirm those results. Huang et al. detected the expressions of matrix metalloproteinase 9 (MMP-9) in meningiomas to determine whether they are a valuable recurrence predictor for meningioma. Increased expressions of MMP-9 were significantly associated with a tumor's microvessel density (MVD), in relation to tumor invasion and tumor recurrence, concluding that together with histological grade increased MMP-9 expression in a tumor might be a valuable predictor for recurrence, especially for benign meningiomas [12]. Also, Kwon et al. showed high MMP-9 expression in clear cell and rhabdoid meningiomas, and high expression of matrix metalloproteinase 9 was associated with tumor recurrence and local invasion at the time of diagnosis [5].

Barresi et al. presented high MMP-9 expression in 46% of meningiomas, and it was significantly correlated with the percentage of progesterone receptor (PR) expression. The recurrence rate was 1.7%. The only recurred case showed high MMP-9 expression, absence of PR, and low Ki-67 LI. They concluded that, in spinal meningiomas, high MMP-9 expression seems to be associated with the development of recurrences only in the absence of PR expression. Thus, the evaluation of both MMP-9 and PR expression might be of use in the identification of spinal meningiomas at higher risk of relapse [2].

In our present study, we also showed a significant correlation between MMP-9 and the presence of peritumoral brain edema; all of the meningiomas with presence of peritumoral brain edema showed strong MMP-9 expression. There is a lack of present data indicating the necessity of MMP-9 investigation in association with PTBE. Only Jung et al. also examined the relationship between the severity of peritumoral edema and the expression of intracystic MMP. They revealed that total and activated MMP-9 levels were significantly associated with the severity of peritumoral edema (*P* < 0.05) [16].

Also Iwado et al. suggested that MMP-9 expression was positively related to VEGF expression and pial blood supply and promoted the occurrence of PTBE by inducing the disruption of the arachnoid membrane and formation of pial blood supply [9]. Paek et al. studied the expression of matrix metalloproteinases (MMP) and tissue inhibitors of matrix metalloproteinase (TIMP) in microcystic meningiomas to investigate a possible underlying mechanism for the development of microcysts and peritumoral edema, which are frequent characteristics of this rare subtype. Compared with the control group, MMP-9 was invariably and highly expressed in immunohistochemical staining of microcystic meningiomas. The results suggested that the increased ratio of MMP-9 to TIMP-1 might be associated with the formation of microcysts and peritumoral edema in microcystic meningioma [17].

Contradictory to those findings, Beltran et al. in a retrospective study investigated the expression of MMP subtypes 9 and 2 in canine intracranial meningiomas and their association with peritumoral edema. They showed that MMP-9 was expressed in all the samples (22/22), whereas proMMP-2 was expressed in 21 of 22 meningiomas, and a/proMMP-2 was expressed in 9 of 22. The immunoreactivity scores were not statistically linked to the severity of peritumoral edema. None of the evaluated MMP expression parameters were statistically linked to the edema index. They concluded that MMP-2 and MMP-9 expression by tumor cells, evaluated through immunohistochemistry, is not predictive of the formation of peritumoral edema in canine rostrotentorial meningiomas [3]. Despite this, all the results suggest that MMPs may be partly involved in the pathogenesis of peritumoral edema in brain tumors. In the present study, we also evaluated VEGF expression in meningiomas of various grades. VEGF is a potential target marker for many types of tumor, including lung or renal cell cancer. In gliomas, VEGF is also a marker associated with poor prognosis and less differentiation. In meningiomas, the results are contradictory. We observed VEGF expression mainly in high-grade meningiomas as well as low-grade meningiomas: angiomatous, secretory, and some of the mixed type ones. VEGF was also statistically significantly correlated with the presence of PTBE.

Similar to our results, Nassehi observed PTBE in 43 out of 101 meningiomas, and the presence of PTBE was correlated with VEGF expression within the tumors [18]. Hou et al. also confirmed the relationship between VEGF expression and PTBE in meningiomas [19]. Markovic et al. showed that PTBE in intracranial meningiomas has significant influence on prognosis in surgically treated patients in terms of increased risk of morbidity and postoperative complications. Similar to our study, they showed that VEGF expression was strongly correlated with PTBE formation, which also affects the outcome in the management of patients with intracranial meningiomas [6].

Lee et al. observed the association between VEGF expression and necrosis in meningiomas and worse clinical outcome [20]. Nassehi showed a positive correlation between VEGF expression and edema concluding that VEGF is responsible for the formation of PTBE and may be a therapeutic target for meningioma treatment [18]. Preusser et al. observed that high microvessel density (MVD) correlates with unfavorable prognosis in a series of recurring meningiomas. In their study, VEGF was frequently expressed in meningiomas and seemed important for tumor growth and recurrence [21]. Jensen and Lee also observed a positive correlation between VEGF expression and PTBE presence in a group of meningiomas. PTBE was also associated with higher grade, larger tumors, and log of MVD. VEGF expression was also correlated with progression-free survival, concluding that VEGF is significantly correlated with tumor recurrence and progression [22]. Salokorpi et al. observed significant VEGF expression in meningioma cases in association with the presence of edema [23].

Other studies also seem to confirm our results. Abdelzaher et al. observed that VEGF expression (38.50% of cases) correlated positively with perifocal edema, tumor size, and proliferative index (PI) [1]. Dharmalingam et al. observed that 65% of Grade I tumors showed VEGF positivity, while 100% of Grade II and Grade III tumors were VEGF positive (*P* = 0.157). There was a gradual increase in microvascular density from tumors which were negative for VEGF to tumors which expressed moderate to strong VEGF. The difference was statistically significant (*P* = 0.009). They concluded that VEGF expression correlated with microvascular density in meningioma irrespective of tumor grade, with a gradual increase in microvascular density in relation to the VEGF score [10].

Markovic et al., in a series of 78 consecutive patients, analyzed the influence of peritumoral edema (PTE) and angiogenesis (vascular endothelial growth factor/VEGF expression) on the prognosis of morbidity and postoperative complications after intracranial meningioma surgery. The severity of PTE showed significant correlation with VEGF expression, and all patients with large PTE (>40 mm) had strong VEGF expression (>50%). Treatment outcome was significantly better in patients with low VEGF expression (*P* < 0.05). All of the monitored postoperative complications were more frequent in the group with PTE. The duration of intensive care treatment in the group with PTE (mean 6.85 days) was significantly longer than in the group without PTE (mean 3.68 days) (*P* = 0.003). In the group without PTE, the outcome was significantly better than in patients with PTE (*P* < 0.01). PTE in intracranial meningiomas had significant influence on prognosis in surgically treated patients in terms of increased risk of morbidity and postoperative complications. VEGF expression was strongly correlated with PTE formation, which also affected the outcome in the management of patients with intracranial meningioma [6].

Pistolesi et al. observed numerous small microvessels (mean: 34) in Grade II-III meningiomas, while the majority of Grade I showed few larger vessels (mean: 13.09) (*P* = 0.000003). Grade II and III meningiomas showed a preponderant expression of VEGF. These results prompt us to speculate that the microvessel pattern could underlie a higher metabolic demand, probably due to a rapid growth with a consequent worse clinical behavior of the tumor. In this sense, the vascular pattern may be used as a prognostic factor in order to mostly focus attention on those Grade I meningiomas which have a higher likelihood of either recurrence or development of peritumoral edema [13].

Meningiomas are still poorly examined tumors, and the predictive factors are less known than in other primary brain tumors. Peritumoral brain edema is one of the most important factors leading to worse postoperative recovery and clinical outcome. Our study seems to prove the significant role of MMP-9 and VEGF expression in the pathogenesis of peritumoral brain edema in low- and high-grade meningiomas as well as in recurrence or malignant transformation. That is why MMP-9 and VEGF may be useful prognostic factors for worse clinical outcome of meningiomas.

## Figures and Tables

**Table 1:** Meningioma grade in correlation with peritumoral brain edema.

	Peritumoral brain edema grade in Steinhoff classification				Total
	0	I	II	III	
Low-grade meningioma (G1)	37 (27,2%)	33 (24,3%)	22 (16,2%)	1 (0,7%)	93 (68,4%)

High-grade meningioma (G2 and G3)	7 (5,1%)	16 (11,8%)	19 (14,0%)	1 (0,7%)	43 (31,6%)

Total	44 (32,3%)	49 (36,1%)	41 (30,2%)	2 (1,4%)	

**Table 2:** MMP-9 and VEGF expression in correlation with meningioma grade.

	Low-grade meningioma (G1) *n* = 93	High-grade meningioma (G2 and G3) *n* = 43	*P* value
MMP-9 expression			**0,003**
(−)	28 (30,1%)	3 (6,9%)	
(+)	65 (69,9%)	**40 (93,1%)**	
VEGF expression			**0,001**
(−)	**65 (69,9%)**	14 (32,6%)	
(+)	27 (29,0%)	**29 (67,4%)**	
(++)	1 (1,1%)	0 (0,0%)	

**Table 3:** MMP-9 expression in correlation with the grade of peritumoral brain edema.

MMP-9 expression	Peritumoral brain edema grade in Steinhoff classification				*P* value
	0	I	II	III	
	*n* = 44	*n* = 49	*n* = 41	*n* = 2	
(−)	14	14	3	0	0,028
(+)	30	**35**	**38**	**2**	

**Table 4:** VEGF expression in correlation with the grade of peritumoral brain edema.

VEGF expression	Peritumoral brain edema grade in Steinhoff classification				*P* value
	0	I	II	III	
	*n* = 44	*n* = 49	*n* = 41	*n* = 2	
(−)	34	22	23	0	**0,005**
(+)	10	**27**	**17**	**2**	
(++)	0	0	**1**	0	
